# Case report and literature review: Misdiagnoses of contrast encephalopathy as stroke

**DOI:** 10.1097/MD.0000000000041473

**Published:** 2025-02-14

**Authors:** Xiangjia Qi, Liqian Gao, Lifeng Qi

**Affiliations:** a Department of Neurology, Liaocheng People’s Hospital, Liaocheng, China.

**Keywords:** carotid artery stenting, complications, contrast encephalopathy, iodinated contrast stroke mimics, iodixanol

## Abstract

**Rationale:**

Contrast-induced encephalopathy (CIE) is a rare complication following carotid artery stenting, often mimicking stroke symptoms such as focal neurological deficits. Its transient nature and reversibility necessitate differentiation from critical complications like cerebral hemorrhage or infarction, as management strategies differ substantially. This case underscores the diagnostic challenges and clinical implications of CIE in endovascular procedures.

**Patient concerns:**

A 65-year-old woman with a history of cerebral infarction and prior carotid artery stenting presented with persistent numbness in her extremities for over 1 month. Post-carotid artery stenting, she developed acute slurred speech and right-sided limb weakness within 1 hour, raising concerns for stroke or procedural complications.

**Diagnoses:**

Emergency cranial computed tomography revealed left cerebral hemisphere swelling and linear hyperdensities, while magnetic resonance imaging demonstrated punctate diffusion-weighted imaging hyperintensities in the left parietal lobe. Temporal correlation of symptoms with iodixanol contrast administration (150 mL) supported a diagnosis of CIE, excluding acute infarction or hemorrhage.

**Interventions:**

Immediate management included antiplatelet therapy (tirofiban), thrombolysis (urokinase), corticosteroids (methylprednisolone), and antiedema agents (mannitol and albumin). Supportive care emphasized hydration and neurological monitoring.

**Outcomes:**

The patient exhibited progressive improvement, with complete resolution of speech and motor deficits by postoperative day 3. Neuroimaging abnormalities regressed, aligning with the transient nature of CIE.

**Lessons:**

This case highlights the critical role of prompt neuroimaging to exclude life-threatening differentials and confirms CIE’s reversibility with supportive care. Risk factors such as contrast volume and cerebral circulation dynamics warrant attention. Clinicians should exercise caution in readministering iodinated contrast to patients with prior CIE episodes.

## 1. Introduction

Hyperperfusion syndrome, embolism, cerebral hemorrhage, plaque detachment, or subarachnoid hemorrhage are frequent complications after carotid artery stenting (CAS).^[1]^ In comparison with the above complications, contrast encephalopathy is a rare complication of CAS.^[1,2]^ Its clinical symptoms resemble that of a stroke, including convulsions, cortical blindness, encephalopathy, and localized neurological abnormalities.^[3,4]^ Therefore, it can be challenging to differentiate contrast-induced encephalopathy (CIE) from other more commonly occurring complications.^[5,6]^

## 2. Case description

A 65-year-old female patient presented to our clinic for evaluation, 1 year after undergoing a right carotid artery stenting procedure. Six years before presentation, the patient had multiple cerebral infarctions in different regions of the anterior circulation of both cerebral hemispheres, resulting in a slight movement impairment on the left limb. During a cerebral angiography procedure conducted 4 years prior, the patient presented with aphasia and right limb hemiplegia, which were promptly relieved by intracatheter arterial thrombolysis. One year before the presentation, the patient experienced aphasia and disturbance of consciousness following the right CAS procedure, but her symptoms improved spontaneously on the second postoperative day. The patient reported no history of hereditary predisposition in her family.

**Figure 1.**
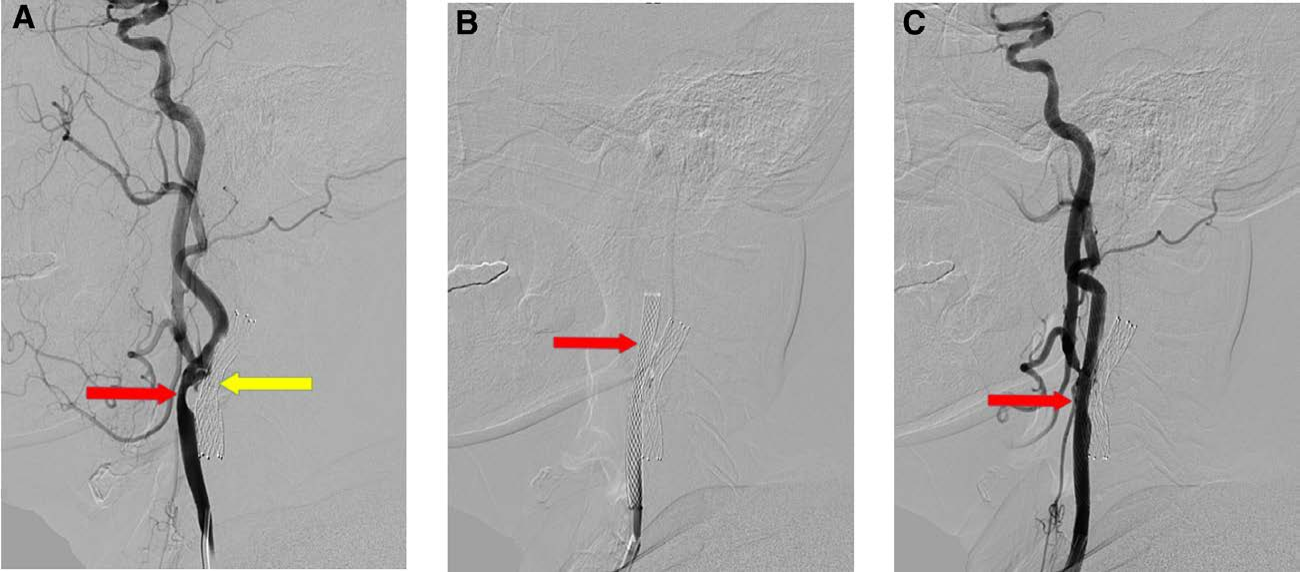
Comparison of patients with carotid artery stenting before and after implantation. (A) The last left general carotid artery stenosis in the DSA. The yellow arrow is the stent shadow of the last general carotid artery stent implantation. (B and C) Unobstructed stent insertion lumen in the DSA. DSA = digital subtraction angiography.

Next, the digital subtraction angiography (DSA) examination suggested severe stenosis of the terminal left common carotid artery and the beginning of the internal carotid artery with a stenosis of ≈80% (Fig. 1A). The North American Symptomatic Carotid Endarterectomy Trial method was used for the grading of stenosis. Hence, we then implanted a 9 × 40 mm^2^ WALLSTENT self-expanding stent at the left carotid artery stenosis site (Fig. 1B and C). During the procedure, we used iodixanol as a contrast agent, with a total volume of ≈150 mL. The preoperative blood pressure of the patient was 122/84 mm Hg, and the intraoperative systolic blood pressure fluctuated at ≈100 to 120 mm Hg. The patient was transferred to the care unit after the procedure, and the measured blood pressure of the patient was 98/53 mm Hg.

**Figure 2.**
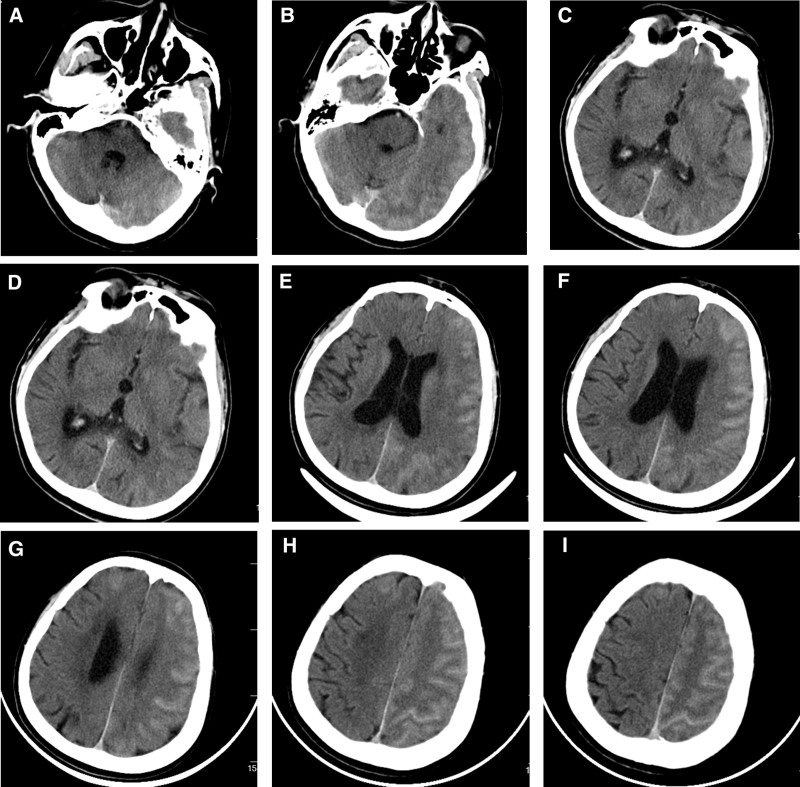
CT scan of the brain immediately after stenting when the patient developed symptoms. (A–I) Multiple high-density strips were observed in the left cerebral hemisphere, part of the right frontal sulcus, cerebral falx, and cerebellar curtain. Edema of brain tissue was also observed. CT = computed tomography.

Approximately 1 hour after the procedure, the patient suddenly presented with confusion, slurred speech, aphasia, and weakness in the right limbs without any accompanying symptoms, such as nausea, vomiting, fever, chills, and limb convulsion. The neurological examination revealed that the muscle strength of the right upper limb and right lower limb was graded 4 and 3, respectively, muscle strength of the left limbs was normal, muscle tone of all limbs was normal, Babinski sign was negative bilaterally, and meningeal irritation sign was negative. The emergency cranial computed tomography (CT) re-examination suggested edema in the large area of left cerebral hemisphere; swelling and blurring of gyri; shallowing of sulci; and cerebral fissures compared with the contralateral side; midline was in the center; and left cerebral hemisphere, some sulci in the right frontal lobe, falx cerebri, and tentorium cerebelli had multiple striped hyperdensities (Fig. 2A–I).

The cranial CT examination suggested that the patient had multiple striped hyperdensities in the sulci of the left cerebral hemisphere, but there was no obvious mass effect, and the density was similar to that of the cerebrovascular lumen. Therefore, we attributed this change in imaging to diffuse extravasation of contrast agents rather than hemorrhagic-related disease. We also ruled out the diagnosis of cerebral hyperperfusion syndrome considering the patient had relatively stable blood pressure levels throughout the perioperative period. Combining the relevant symptoms and physical signs of the patient, we consider the diagnosis of either cerebral arterial embolism or contrast encephalopathy to be the most likely explanation. Therefore, we immediately administered 300,000 U of urokinase and tirofiban empirically through continuous intravenous pumps. On the same day, we administered 10 g of human albumin and 40 mg of methylprednisolone sodium succinate intravenously.

**Figure 3.**
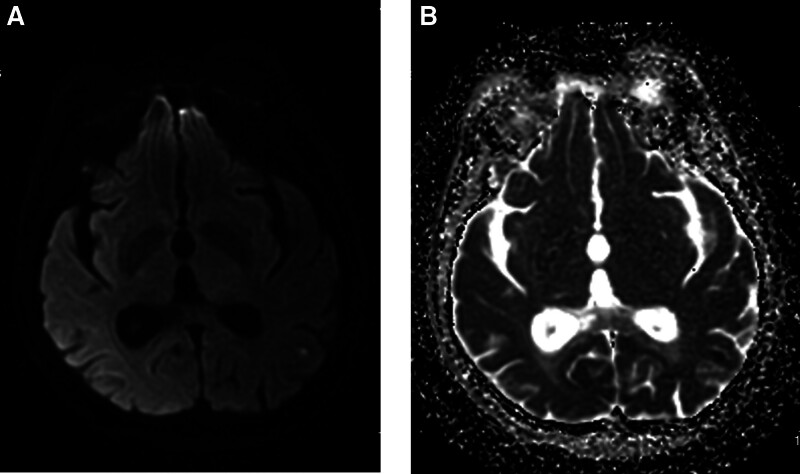
Follow-up cranial MRI after CAS. (A) Magnetic resonance DWI imaging after CAS, DWI point-like high signal in the left parietal lobe, with possible acute/subacute cerebral infarction. (B) Magnetic resonance ADC imaging after CAS. ADC = apparent diffusion coefficient, CAS = carotid artery stenting, DWI = diffusion-weighted imaging.

On the first postoperative day, magnetic resonance imaging was performed on the patient and the results showed a punctate diffusion-weighted imaging high signal in the left parietal lobe (Fig. 3A and B). In addition, the previously observed multiple striped hyperdensities in the left cerebral hemisphere also significantly dissipated, which further ruled out the diagnosis of hemorrhagic-related disease. However, this punctate infarction could not explain the series of clinical symptoms experienced by the patient at this moment. At the same time, the patient’s symptoms did not improve following the administration of urokinase, leading us to rule out the diagnosis of cerebral artery embolism. The diagnosis of CIE was established as a result. We immediately discontinued tirofiban and urokinase and continued to administer the same dose of human albumin and methylprednisolone sodium succinate as was given on the day of the procedure, with an additional 150 mL of mannitol injection intravenously. On postoperative day 2, we administered the same medication to the patient as the previous day, resulting in the complete resolution of all clinical symptoms.

After the patient’s symptoms improved and she was discharged, we conducted regular follow-up calls with her, and no further symptoms, such as aphasia or hemiplegia, were observed.

## 3. Discussion

In this case report, we described an elderly woman who presented with confusion, slurred speech, and progressive reduction in the right limb muscle strength immediately after CAS. Our poor knowledge of patients with CIE coupled with its nonspecific clinical presentation posed a challenge to our diagnostic and treatment process. We promptly completed the relevant imaging examinations. After excluding other possible diagnoses, the patient was diagnosed with CIE based on the sudden onset of symptoms, clinical manifestations, neurological examination, and relevant imaging manifestations. We administered a combination therapy such as cortisol-like hormones and administered medications to reduce brain tissue edema, and the patient responded well to this treatment regimen and achieved a clinical cure on postoperative day 2. Throughout the treatment, the differential diagnosis should include embolism secondary to stenting, stent thrombosis, carotid artery dissection, and cerebral hemorrhage due to hyperperfusion after stenting. Among these, good patient outcomes and rapid recovery are the key points to guide clinicians in diagnosing CIE.

The patient underwent multiple surgical procedures using the same iodinated contrast agent, iodixanol, which resulted in clinical presentations mimicking strokes. This has not been reported previously in the literature. This suggests that the occurrence of CIE may be related to the concentration of contrast agents in the cerebral circulation rather than the type.^[7]^ However, there are also those who contend that CIE is treated as an idiosyncratic reaction to the contrast agent, rather than as a side effect related to dose.^[8]^ In a case report by Li et al,^[9]^ they reported on a patient who underwent 2 brain DSA procedures within a week. However, neurological complications occurred only after the second DSA procedure. In addition, due to the nonspecific clinical presentation of CIE and the exclusive diagnostic methods, there is a high possibility of misdiagnosis. Timely imaging examinations are particularly important in the diagnostic process.

The CIE associated with CAS is rarely reported in the literature, and most of them were case reports of a single case. To date, there have been 5 cases diagnosed with CIE following CAS (Table 1). During the procedure, a nonionic-iodinated contrast agent was used in 3 cases, an ionic-iodinated contrast agent was used in 1 case, and the use of a contrast agent was not mentioned in 1 case. In terms of clinical symptoms, 4 cases presented with varying degrees of unilateral limb weakness, 3 cases presented with aphasia, and other symptoms included neglect or impaired consciousness. In all 5 cases, a plain cranial CT scan was performed after the patients developed relevant clinical symptoms, except for 1 case in which no abnormality was observed. The majority of the patients presented with edema in the treated cerebral hemisphere or imaging manifestations similar to subarachnoid hemorrhage. In terms of treatment regimen, in addition to close clinical monitoring and supportive therapy, 3 cases were administered with mannitol or albumin to dehydrate brain tissues, 2 cases were given no special treatment, and other treatment modalities included steroids, such as dexamethasone and methylprednisolone. It is reassuring to note that all 5 patients achieved clinical cure in 2 days to 2 weeks without any residual symptoms. The patient we report here experienced aphasia multiple times after the injection of a contrast agent, which seemed to be unrelated to the site of injection. In addition, the duration of the patient’s symptoms appeared to be related to the amount of contrast agent used.

**Table 1:** Cases of post-CAS contrast encephalopathy reported thus far in the literature.

References	Procedure	Type of contrast	Symptoms	Imaging findings	Medical treatment	Clinical resolution
Jiang et al^[10]^	DSA + CAS	Unknown	Paralysis of the right upper limb, unclear speech, fever, and restlessness	CT: swelling and a linear high-density area in the left cerebral hemisphere	Dexamethasone Mannitol Albumin	1 wk
Dangas et al^[11]^	DSA + CAS	Ioxaglate (ion)	Confusion, left hemiparesis, neglect	CT: right frontoparietal cortical enhancement and edema	Not available	2 d
Fang et al^[12]^	PCI + CAS	Iomexol (nonion)	Right hemiparesis	CT: diffuse cortical enhancement similar to subarachnoid hemorrhage	Not available	7 d
Menna et al^[13]^	DSA + CAS	Iomeron (non)	Aphasia	Normal CT and MRI	Mannitol Hydration	2 wk
Chisci et al^[7]^	PCI + CAS	Iodixanol	Aphasia, stupor, and hemiparesis	CT: left hyperdensity of some subarachnoid spaces	Mannitol Methylprednisolone	2 d

CAS = carotid artery stenting, CT = computed tomography, DSA = digital subtraction angiography, MRI = magnetic resonance imaging, PCI = percutaneous coronary intervention.

The etiology of CIE remains elusive to this day. It is suggested that the ionic properties and high osmotic pressure of contrast agents may temporarily compromise the integrity of the blood–brain barrier, resulting in the infiltration of the contrast agents into the central nervous system and inducing direct neurotoxicity. However, the administration of nonionic and iso-osmolar contrast agents can also lead to similar consequences.^[14,15]^ In addition, high-pressure and rapid injection of contrast agents can alter the hemodynamics of cerebral blood flow, potentially leading to cerebral vasospasm and interruption of microcirculation.^[16,17]^

Due to the uncertainty and rarity of CIE, it is difficult to establish specific recommendations beyond the usual precautions that apply to general patients, which are using the lowest possible dosage of contrast agents and adequate hydration throughout the procedure. In most patients diagnosed with CIE, only supportive treatment is required, including appropriate intravenous crystalloid fluid and anticonvulsants, and the neurological status will gradually return to baseline levels. In few cases where steroids and mannitol-based drugs were given to the patients, there were no serious side effects.^[7,18,19]^ In patients undergoing treatment with glucocorticosteroids, therapy was initiated only after excluding other potential diagnoses, and a high suspicion of CIE was established. However, the literature has not yet provided specific recommendations regarding the dose of steroids and when to discontinue them.^[20,21]^ In our opinion, the use of steroids should be personalized and based on a comprehensive evaluation of the patient’s clinical symptoms and the type and dose of the contrast agent used.

Since it was first reported as transient cortical blindness after coronary angiography by CIE in 1970,^[22]^ the vast majority of patients in subsequent related case reports have had reversible clinical symptoms. In the cerebrovascular field, 2 cases of irreversible visual field defects^[23,24]^ and 1 case of being left with a mild dysarthria and a mild right monoparesis^[25]^ were reported, both of which occurred after aneurysm embolization. Also, Zhao et al^[8]^ reported a case of patient death due to irreversible cerebral edema after cerebral arteriography.

## 4. Conclusion

CIE is an extremely rare complication during both cerebral angiography and CAS. Due to its extremely low incidence and diverse clinical manifestations, it poses challenges in clinical diagnosis and treatment. Timely brain imaging is the key to rule out other similar diagnoses and is also crucial to the subsequent treatment regimen. Therefore, the risk-to-benefit ratio for using iodinated contrast agents in patients should be thoroughly evaluated preoperatively, especially for those patients with a previous diagnosis of CIE or in whom CIE cannot be ruled out.

## Acknowledgments

The authors highly appreciate the understanding and support from the patient and her family.

## Author contributions

**Writing – original draft:** Xiangjia Qi.

**Writing – review & editing:** Xiangjia Qi.

**Data curation:** Liqian Gao.

**Formal analysis:** Liqian Gao.

**Resources:** Liqian Gao.

**Conceptualization:** Lifeng Qi.
